# Anticryptococcal Evaluation of the Allylimine 3H2: Modulation of Virulence Traits and Synergistic Action with Amphotericin B

**DOI:** 10.1021/acsomega.6c01027

**Published:** 2026-04-27

**Authors:** Thais Furtado Ferreira Magalhães, Heber Victor Tolomeu, Luíza Braga Ferreira dos Santos, Cleide Viviane Buzanello, Danielle Letícia Silva, Gabriella Freitas Ferreira, Julliana Ribeiro Alves dos Santos, Marliete Carvalho da Costa, Vanessa da Silva Dutra de Carvalho, Daniel Assis Santos, Cleiton Moreira da Silva, Maria Aparecida de Resende-Stoianoff, Ângelo de Fátima

**Affiliations:** † Departamento de Microbiologia, 28114 Universidade Federal de Minas Gerais, Belo Horizonte, MG 31270-901, Brazil; ‡ Departamento de Química, Universidade Federal de Minas Gerais, Belo Horizonte, MG 31270-901, Brazil; § Departamento de Ciências Ambientais e Ciências Aplicadas À Saúde, 74346 Universidade Estadual do Oeste do Paraná, Toledo, PR 85919-110, Brazil; ∥ Departamento de Farmácia, Universidade Federal de Juiz de Fora, Campus Governador Valadares, Governador Valadares, MG 35010-180, Brazil; ⊥ Universidade de Pernambuco, Instituto de Ciências Biológicas, Recife, PE 50100-010, Brazil; # Instituto de Biología Funcional y Genómica, Universidad de Salamanca - Consejo Superior de Investigaciones Científicas, Salamanca 37008, Spain; ∇ Programa de Pós-graduação em Biociências Aplicada À Saúde, Universidade CEUMA, São Luís, MA 65075-120, Brazil; ○ Institute of Chemistry and Biochemistry, Freie Universität Berlin, Berlin 14195, Germany; ◆ Department of Organic Chemistry, Faculty of Science, Atatürk University, Erzurum 25240, Türkiye

## Abstract

The development of new antifungal scaffolds is critical for expanding therapeutic options against cryptococcosis. Here, we evaluated the allylimine 3H2 as a potential lead compound against *Cryptococcus gattii*. The allylimine 3H2 exhibited consistent *in vitro* activity with a geometric mean MIC of 7.5 μg mL^–1^ across 12 strains and showed a synergistic interaction with amphotericin B, with FICI values reaching 0.5. The compound impaired major virulence determinants, reducing capsule size, melanin synthesis, and laccase activity while altering the negative surface charge. In a murine model, 3H2 potentiated amphotericin B therapy, extending survival to over 100 days and markedly lowering fungal burden in both lungs and brain. Docking simulations supported interactions of 3H2 with targets involved in melanin biosynthesis and cell wall-associated processes. These findings highlight 3H2 as a promising scaffold for antifungal drug discovery and support its further optimization as a candidate for combination therapy against cryptococcosis.

## Graphical abstract

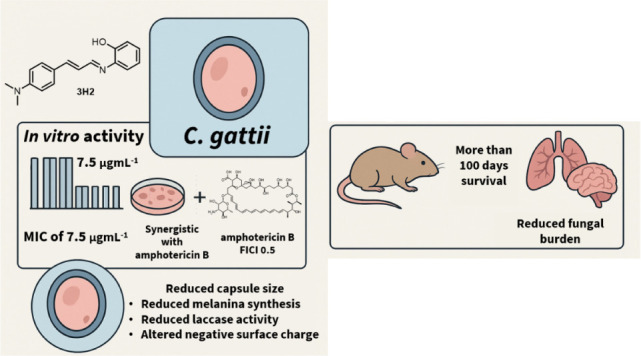

## 1 Introduction

Cryptococcosis is an invasive mycosis caused by yeasts of the genus *Cryptococcus*, with *Cryptococcus gattii* emerging as an important human pathogen. In contrast to *C. neoformans*, which typically affects immunocompromised patients, *C. gattii* can infect immunocompetent individuals, contributing to a substantial disease burden^1,2^ (Figure 1).

**Figure 1:**
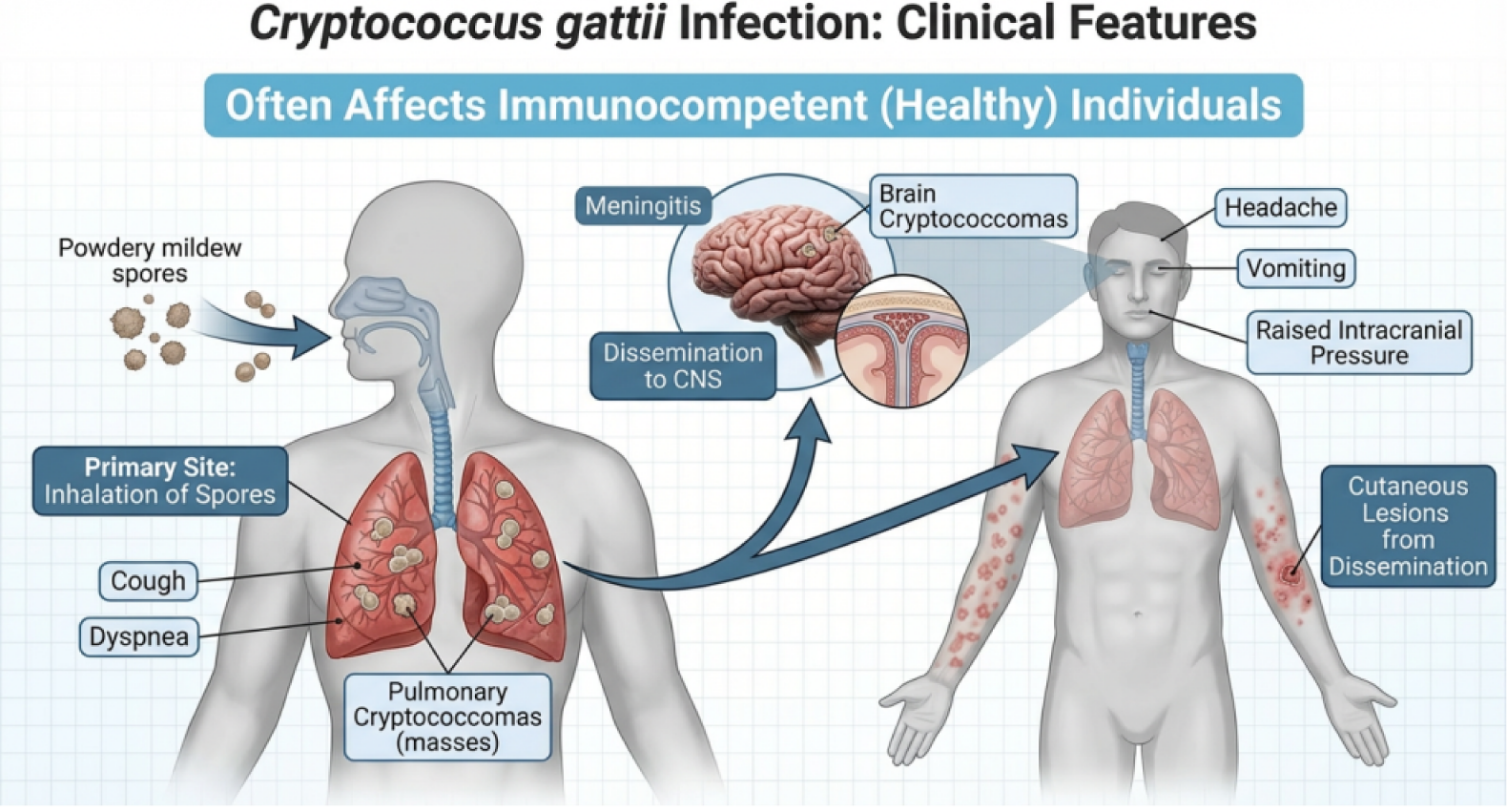
Infection pathway and clinical features of *Cryptococcus gattii*. The diagram illustrates the primary site of infection (lung) via spore inhalation, followed by dissemination to the central nervous system (CNS) and other sites, such as the skin. Key clinical hallmarks, including cryptococcomas, meningitis, and raised intracranial pressure, are highlighted. Image generated with the assistance of artificial intelligence using ChatGPT (OpenAI)-Version 5.3.

Epidemiologically, the prevalence of *C. gattii* infections is estimated to range from 11% to 33% of global cryptococcosis cases, depending on the region and populations studied.^1^ In addition, the severity of disease caused by *C. gattii* is considerable, with high mortality rates and frequent neurological complications, underscoring the need for new therapeutic approaches.^3^ The conventional treatment of *C. gattii* cryptococcosis consists of three phases: (i) induction therapy with amphotericin B (**1**), usually combined with 5-flucytosine (**2**); (ii) consolidation therapy with azoles, such as fluconazole (**3**); and (iii) maintenance therapy (Figure 2).^4^

**Figure 2:**
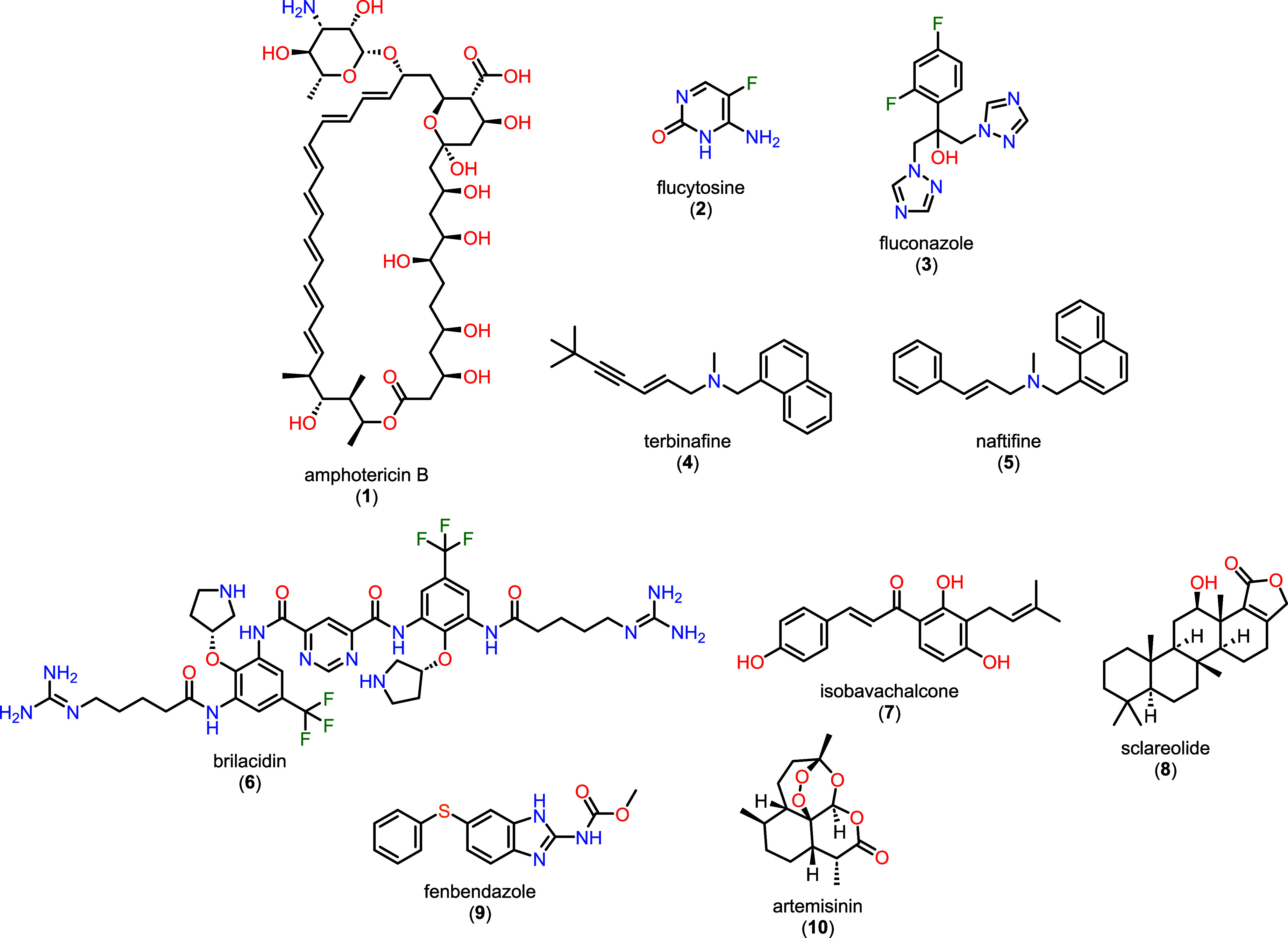
Chemical structures of amphotericin B (**1**), flucytosine (**2**), fluconazole (**3**), terbinafine (**4**), naftifine (**5**), brilacidin (**6**), isobavachalcone (**7**), sclareolide (**8**), fenbendazole (**9**), and artemisinin (**10**).

However, these therapies present substantial limitations, including amphotericin B-associated toxicity, emerging resistance to azoles, limited accessibility to 5-flucytosine in many countries,^5^ and the requirement for prolonged treatment.^6^ Clinical reports have already documented therapeutic failures, including cases of *C. gattii* exhibiting partial resistance to fluconazole.^7,8^ Recent studies have explored novel antifungal compounds and screening strategies against *Cryptococcus* species including antimicrobial peptides, natural products, and drug-repurposing candidates. Several compounds, such as brilacidin (**6**), isobavachalcone (**7**), sclareolide (**8**), fenbendazole (**9**), and artemisinin (**10**) (Figure 2), have shown promising antifungal activity and mechanistic insights in recent investigations.^9–13^ From a biological perspective, *C. gattii* exhibits well-characterized virulence factors that contribute to its pathogenicity. These include the polysaccharide capsule, melanin production via laccase activity, and thermal adaptation.^14,15^ Additionally, genetic analyses have shown that different *C. gattii* genotypes (for example, VG variants) exhibit variable expression of these factors, which may influence their virulence and resistance.^16^ Given these challenges, there is growing interest in therapeutic strategies that extend beyond classical antifungal agents. The identification of specific molecular targets, combined with structure-based drug design, provides a promising pathway for the discovery of innovative antifungal drugs.^17^ Virulence factors play a central role in the pathogenesis of *Cryptococcus*, including the polysaccharide capsule, the ability to grow at 37 °C, metabolic adaptation, and, of particular relevance, the production of melanin (**16**) mediated by the laccase enzyme.^18,19^ Laccase oxidizes DOPA (**611**) to DOPA-quinone (**12**), which subsequently undergoes intramolecular nucleophilic attack by the amino group to generate cyclo-DOPA (**13**).^20^ This intermediate is further transformed into DOPA-chrome (**14**) and then into dihydroxyindole (**15**) derivatives (DOIs) (Figure 3).

**Figure 3:**
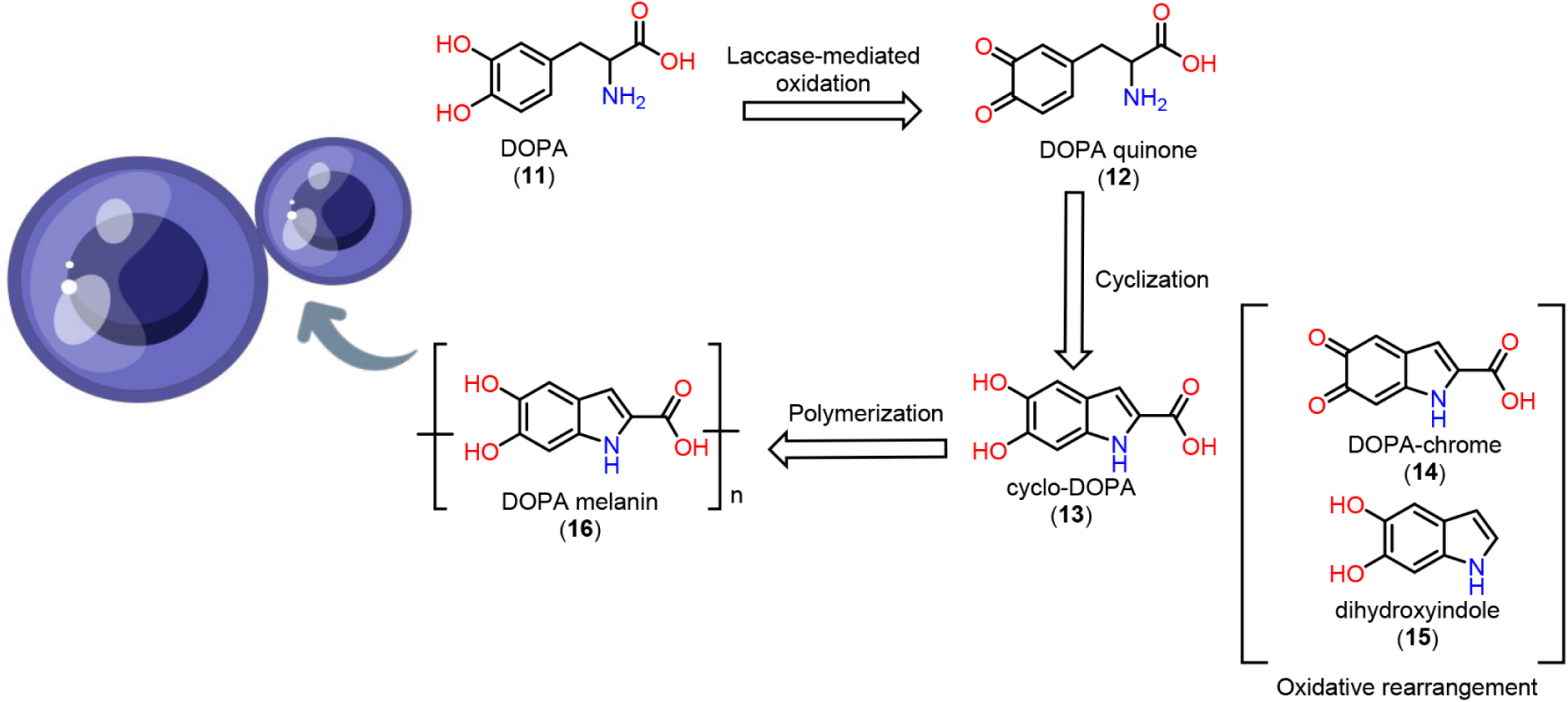
DOPA pathway biosynthesis of melanin and its production model.

Laccase catalyzes the oxidation of phenolic substrates, leading to the formation of melanin, which shields the fungus from oxidative stress, ultraviolet radiation, and antifungal activity. Strains lacking functional laccase exhibit markedly reduced virulence in murine models, underscoring the enzyme’s critical contribution to pathogenicity.^21^ Additional evidence indicates that laccase is associated with the cell wall, where it modulates host–pathogen interactions and facilitates dissemination to the central nervous system.^21^ Moreover, the expression of the *LAC1* gene is regulated by signaling pathways such as cAMP/PKA, which also coordinate capsule formation and stress adaptation, further emphasizing its integration within the broader virulence network.^22^ The pharmacological interest in laccase as a therapeutic target has increased, as inhibitors of this enzyme reduce melanization, enhance fungal susceptibility, and attenuate virulence. Several small phenolic and heterocyclic molecules have been shown to inhibit laccase activity, markedly reducing melanin formation in *Cryptococcus* and exhibiting adjunctive antifungal effects.^23^ Laccase inhibitors have also exhibited synergy with amphotericin B in experimental models, further supporting their potential as an emerging target for combination therapies.^24^ In this context, there is growing interest in molecules capable of simultaneously interfering with classical fungal survival pathways and virulence determinants. Allylamine compounds, such as terbinafine (**4**) and naftifine (**5**) (Figure 2), belong to a class of antifungals that inhibit squalene epoxidase, an essential enzyme in the ergosterol biosynthetic pathway.^25^ They have already demonstrated antifungal activity and support the chemical exploration of related scaffolds.^25^

Thus, related synthetic compounds, such as 3H2 (**17**) (Figure 4), have emerged as promising candidates for virulence modulation, inhibition of melanization, and potential enhancement of the therapeutic efficacy of existing antifungal agents. Accordingly, the aim of the present study is to investigate the antifungal activity of compound 3H2 against *C. gattii*, to characterize its effects on capsule formation, melanization, laccase activity, and zeta potential; to evaluate its interaction with amphotericin B; and to employ molecular docking to explore potential targets and structural mechanisms associated with its activity.

**Figure 4:**
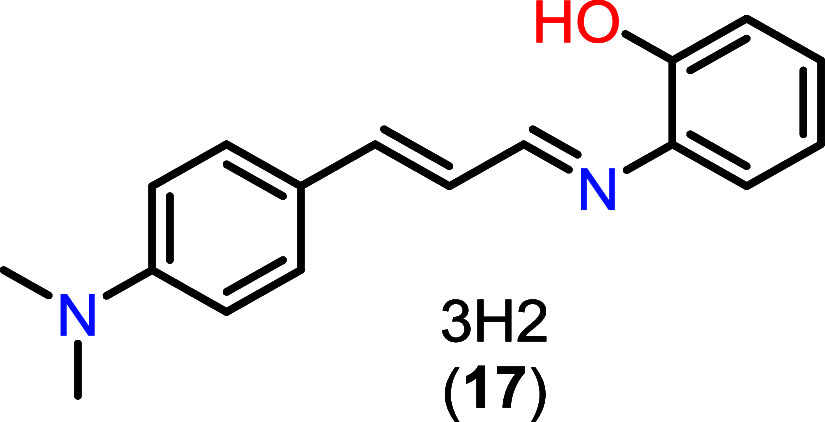
Chemical structure of 3H2 (**17**).

## 2 Experimental Section

### 2.1 Chemistry

Compound 3H2 was prepared through the condensation of 4-dimethylaminocinnamaldehyde with 2-aminophenol under microwave irradiation, leading to the formation of a Schiff base. The reagents were used in equimolar amounts and dissolved in absolute ethanol before exposure to microwave heating in a CEM Discover reactor for 2 min. The course of the reaction was followed by thin-layer chromatography. Upon completion, the mixture was cooled to room temperature, and the crude material was purified by crystallization from ethanol to provide the allylimine product in 80% yield (Figures S1 and S2).

### 2.2 In Vitro

#### 2.2.1 Antifungal Susceptibility

Initially, to determine the anticryptococcal activity of 3H2, the minimum inhibitory concentrations (MICs) against 12 *Cryptococcus gattii* strains were determined according to Clinical and Laboratory Standards Institute (CLSI) guidelines.^26^ The concentrations tested ranged from 0.25 to 128 μg mL^–1^ for 3H2, and for the positive controls fluconazole (FCZ) 0.125 to 64 μg mL^–1^, and amphotericin B (AMB) 0.03 to 16 μg mL^–1^. The stock solutions of 3H2 and AMB, used as a positive control, were prepared in dimethyl sulfoxide (DMSO; Sigma-Aldrich, St. Louis, MO), and the final solvent concentration used in the assay was 1%. The control compound FCZ was prepared in sterile distilled water. Growth, sterility, and toxicity controls were included on all of the plates. The minimal inhibitory concentration (MIC) was defined as the concentration that produced no visible growth for 3H2 and AMB, and as a 50% growth inhibition for FCZ, after 72 h of culture at 35 °C. After the MIC determination, the minimum fungicidal concentrations (MFCs) were determined by plating out 0.1 mL of suspension from each well showing inhibition of growth (MIC, MICX2, and MICX4) onto Sabouraud dextrose agar and incubating at 35 °C. The MFC was defined as the lowest drug concentration at which no colonies were observed after 72 h of culture.^27,28^

#### 2.2.2 Synergism Study between 3H2 and Fluconazole or Amphotericin B against *C. gattii* Strains (Checkerboard Assay)

The combination of 3H2 + FCZ and 3H2 + AMB against *C. gattii* strains was evaluated using the checkerboard assay.^29^ The fractional inhibitory concentration index (FICI) was calculated by the sum of the FICs for each drug; the FIC is defined as the MIC of each drug when used in combination divided by the MIC of the drug when used alone. The synergism study between these drugs was classified as synergism if FICI was *<*0.5, no interaction if 0.5 *<* FICI *<* 4, and antagonism for FICI *>*4.^30^

#### 2.2.3 Morphometric and Zeta Potential Analysis

*C. gattii* L27/01 yeast cells were grown on minimal medium agar (15 mM glucose, 10 mM MgSO_4_, 29.4 mM KH_2_PO_4_, 13 mM glycine, 3.0 μM vitamin B1, and 2% agar) plates containing 0.5 MIC of 3H2 for 48 h at 37 °C. A no-drug plate was used as a negative control. India ink preparations were made and viewed with an optical microscope (400X) Nikon (Eclipse E-200), and pictures were taken with a Nikon Coolpix 4500 camera. Cell and capsule sizes of 30 yeasts were analyzed and measured using *image J* NIH morphometry software version 1.48v (National Institutes of Health, NIH, Bethesda, MD, United States).^31^ The zeta potentials of suspensions of yeast cells treated with 3H2 (8, 16, and 24 μg mL^–1^were measured with a Zetasizer Nano ZS90 zeta potential analyzer (Malvern Panalytical, Malvern, United Kingdom) as previously described.^32^

#### 2.2.4 Melanin Production and Laccase Activity

To determine whether 3H2 could impact the melanization of the *C. gattii* L27/01 strain, the compound was added at 0.5 MIC and MIC concentrations to minimal medium agar supplemented with 1 mM l-dopa (Sigma, St. Louis, MO). Then, 0.01 mL of a 10^5^ cells mL^–1^ yeast suspension prepared in sterile saline was spotted onto the medium. The cultures were incubated at 30 °C for 10 days and examined daily for growth and melanin production. To investigate the effect of the drug on laccase enzyme activity, a quantitative assay using the oxidation of 2,2-azinobis­(3-ethylbenzothiazoline-6-sulfonic acid) (ABTS; Sigma) as a substrate was performed. *C. gattii* L27/01 yeast was grown in asparagine medium (asparagine 1 g L^–1^, 10 mM sodium phosphate [pH 6.5], MgSO_4_ 0.25 g L^–1^, CuSO_4_ 10 μM) with glucose (1.5 g L^–1^) for 72h at 30 °C. The cells were collected by centrifugation, and a suspension containing 1 × 10^8^ cells mL^–1^ was prepared in PBS with or without 3H2 at 0.5 MIC. The ABTS solution (10 mM) was diluted 1:10 in fungal suspensions with or without treatment, and the tubes were incubated for 2 h at 30 °C. Then, the cells were removed by centrifugation, and the absorbance readings of the solutions were measured at 420 nm. A laccase solution produced commercially (Sigma, 51639) at 1 U mL^–1^ in PBS was used as a positive control, and the negative control was ABTS alone. The test was also conducted by incubating the commercial laccase with different concentrations of 3H2.^33^

### 2.3 *In Vivo*Studies

#### 2.3.1 Ethics, Intratracheal Infection, Survival Curve, Behavioral Analysis and Fungal Burden

The protocol for animal studies was approved by the Comissão de Ética no Uso de Animais (CEUA) of the Universidade Federal de Minas Gerais, Minas Gerais, Brazil (protocol 11/2013). A murine model of pulmonary cryptococcosis with female C57BL/6 (16-18 g) aged 6 to 8 weeks old, was used to investigate the therapeutic effect of 3H2 alone and in combination with amphotericin B. The mice (six per cage) were housed in clean bedding with food and water *ad libitum,* in a controlled environment with a 12h light/dark cycle. For intratracheal infection, mice were anaesthetized intraperitoneally (ip) with ketamine (80 mg kg^–1^) and xylazine (10 mg kg^–1^). Next, they were inoculated intratracheally with 30 μL *C. gattii* strain L27/01 at 10^4^ CFU or PBS (NI group). Mice were divided into different groups, and the treatments began 24 h after fungal inoculation, administered once a day by i.p. injection, as follows: (1) 3H2 (30 mg kg^–1^); (2) AMB (0.5 mg kg^–1^); (3) 3H2 (30 mg kg^–1^) + AMB (0.5 mg kg^–1^); (4) nontreated (NT); and (5) NI.^34^ The suspension for 3H2 administration was prepared by dissolving it in a mixture of surfactants and cosolvents containing PBS (80%), Kolliphor EL (10%), PEG 400 (5%), and propylene glycol (5%).^35^ Amphotericin B solution was prepared in water for injection. Animals were monitored daily for survival and had their behavior analyzed using the SmithKline/Harwell/Imperial College/Royal Hospital/Phenotype Assessment (SHIRPA) protocol, which evaluates five functional categories: neuropsychiatric state, motor behavior, autonomous function, muscle tone and muscular strength, and reflex and sensorial function. The score of each functional category was calculated as the sum of the parameters evaluated and was analyzed using EpiData 3.1.^34,36^ The fungal burdens of additional groups of animals were also evaluated. Mice were euthanized 15, 30, and 130 days after fungal inoculation, and their lungs and brain were aseptically removed, weighed, homogenized, diluted in PBS, plated on SDA, and incubated for 48 h at 37 °C.^34,37^

### 2.4 Statistical Analysis

Statistical analyses were performed using GraphPad Prism, version 5.00, for Windows (GraphPad Software, San Diego, CA, USA), with *P* <.05 considered to indicate significance. Survival curves were plotted using Kaplan–Meier analysis, and results were analyzed using the log-rank test. SHIRPA data were analyzed using analysis of variance (ANOVA) and the Newman-Keuls test. The results of capsule size and laccase activity were analyzed by Student’s t test ANOVA. For zeta potential, ANOVA was used, followed by Dunn’s multiple comparison test.

### 2.5 Docking Analysis

For the docking analysis, the crystal structure of Laccase (PDB ID: 1KYA) was used. Based on the cocrystallized ligand, the binding site was defined using a 10 Å radius. A redocking procedure of the cocrystallized ligand was carried out to determine the most suitable scoring function available in GOLD 2022.3.0, considering the RMSD values. The scoring function selected for Laccase was GoldScore.

## 3 Results and Discussion

### 3.1 Chemistry

Microwave-assisted condensation of 4-dimethylaminocinnamaldehyde with 2-aminophenol rapidly generated compound 3H2, corresponding to the expected allylimine, after 2 min of irradiation. The process proceeded in a clean fashion, and the desired imine was obtained directly after recrystallization without requiring further purification.

Structural confirmation was achieved by ^1^H and ^13^C NMR spectroscopy. The ^1^H NMR spectrum displayed the characteristic azomethine proton (HC=N) as a doublet at δ = 8.37 ppm. In the ^13^C NMR spectrum, the imine carbon (C=N) was observed at δ 161.29 ppm, in the range commonly described for conjugated imine systems. These spectroscopic signatures corroborate the formation of the Schiff base linkage and the conjugated electronic structure of the cinnamyl moiety.

### 3.2 3H2 Reduces the Growth of *C. gattii* Strains Alone and in Combination with Amphotericin B

All strains were susceptible to the tested compound, with a MIC geometric mean of 7.5 μg mL^–1^, and an MFC of 65.8 μg mL^–1^. The susceptibility profile was variable among the strains, and the 3H2 concentration used to inhibit the growth of *C. gattii* L27/01 was 4-fold lower than that of fluconazole. The strains were also susceptible to the antifungal drugs used as positive controls. The association of 3H2 with fluconazole was indifferent, but when the compound was tested in combination with amphotericin B, it showed synergism for one-third of the *C. gattii* strains studied (Table 1). These results are consistent with those reported in other studies, which show that the outcome of antimicrobial combinations are strain-dependent.

**Table 1:** Minimal Inhibitory Concentration (MIC), Minimal Fungicidal Concentration (MFC) Values of 3H2 and the Antifungal Drugs Fluconazole (FCZ), Amphotericin B (AMB), and Their Combination against 12 *C. gattii* Strains^a^

	MIC/MFC (μg mL^–1^)						Combination (FICI interpretation)			
*C. gattii*strains	3H2		FCZ		AMB		3H2 + FCZ		3H2 + AMB	
ATCC 24065	8.0	16.0	4.0	64.0	0.3	0.4	1.2	(I)	0.5	(S)
ATCC 32608	11.3	64.0	16.0	32.0	0.5	0.8	2.0	(I)	0.6	(I)
135L/03	16.0	64.0	8.0	45.3	0.4	0.6	1.2	(I)	0.4	(S)
L28/02	5.7	90.5	11.3	64.0	0.6	1.0	1.2	(I)	0.8	(I)
23/10993	4.0	32.0	4.0	22.6	0.5	0.7	1.3	(I)	0.9	(I)
196L/03	5.7	90.5	16.0	32.0	0.5	0.6	1.2	(I)	0.6	(I)
1913/ER	5.7	64.0	8.0	16.0	0.5	0.6	1.2	(I)	0.6	(I)
547 OTTI	11.3	128.0	16.0	128.0	0.4	0.6	1.1	(I)	0.5	(S)
L27/01	8.0	90.5	32.0	64.0	0.3	0.5	0.7	(I)	0.8	(I)
LMM 818	5.7	90.5	11.3	32.0	0.5	0.7	1.8	(I)	0.6	(I)
L24/01	11.3	128.0	8.0	64.0	0.4	0.6	0.7	(I)	0.5	(S)
29/10893	5.7	45.2	4.0	22.6	0.3	0.6	1.2	(I)	0.9	(I)
Geometric mean	7.5	65.8	9.5	41.2	0.4	0.6	1.18		0.62	

a S, synergism (FICI < 0.5); I, indifferent (0.5 < FICI < 4).

### 3.3 Capsule Volume, Zeta Potential, Melanin Production, and Laccase Activity are Influenced by the Treatment with 3H2

When *C. gattii* L27/01 cells were incubated with 3H2 at 0.5× MIC, their capsule volume was reduced compared to the no-treatment group (*P* < 0.001) (Figure 5). In addition, the zeta potential was higher when the cells were treated with different concentrations of the compound (*P* < 0.0001) (Figure 4).

**Figure 5:**
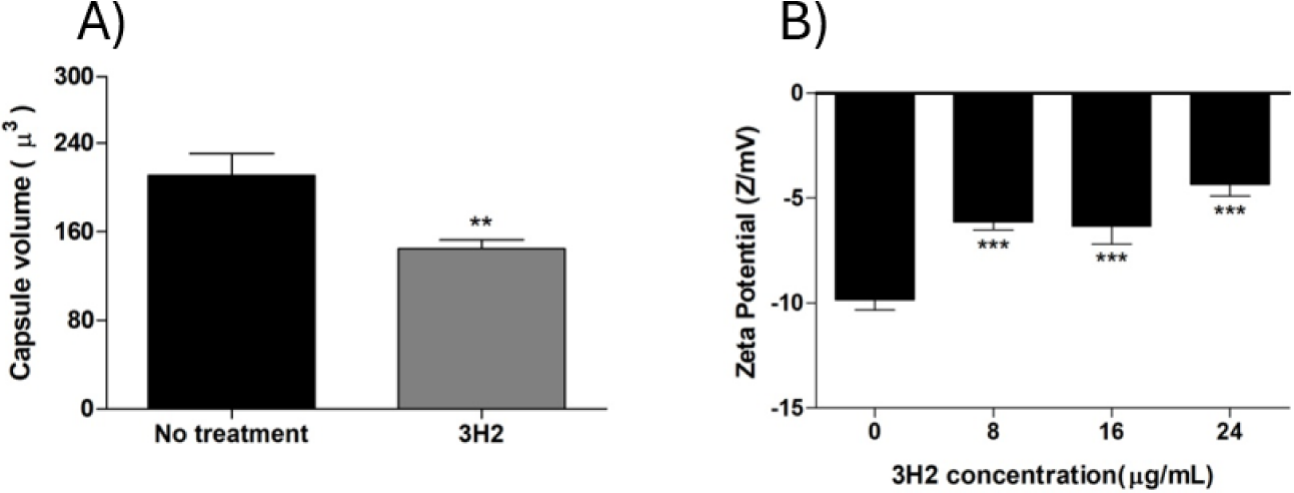
Treatment with 3H2 reduces the capsule volume (A) and zeta potential (B) of *C. gattii* L27/01 cells. **P < 0.01; ***P < 0.001.

It was also possible to visualize the reduction in melanin production of treated cells, which was confirmed by the decreased capacity of laccase to oxidize ABTS (*P* < 0.0001) (Figure 6).

**Figure 6:**
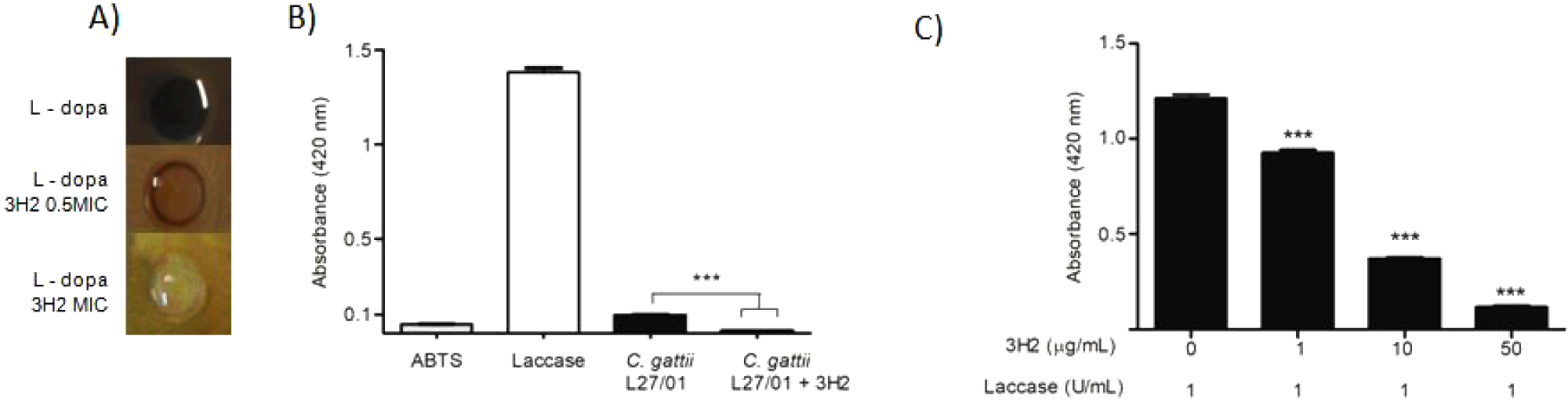
*C. gattii* cells are affected by 3H2 in both melanin production and laccase activity. *C. gattii* L27/01cells were grown on minimal medium 1 mM l-dopa agar plates for 10 days at 30 °C with or without the addition of 3H2 (A). Oxidation of ABTS by *C. gattii* laccase is reduced by 3H2 (B). Increasing concentrations of 3H2 were observed with a constant amount of commercial laccase (C). (***P < 0.0001).

### 3.4 The Association of 3H2 with Amphotericin B Increases Survival, Reduces Fungal Burden and Reduces the Morbidity of the Cryptococcosis Murine Model

Initially, we tested four different doses (30, 50, 100, and 150 mg kg^–1^) of 3H2 for the treatment of murine cryptococcosis, and all showed no increase in the survival of the mice (data not shown). Therefore, the lowest concentration (30 mg kg^–1^) was selected to be investigated in association with amphotericin B. The mice infected with *C. gattii* L27/01 that had not received any treatment (NT) and those treated with 3H2 in monotherapy survived for 19 and 20 days, respectively (Figure 7). The treatment with AMB (0.5 mg kg^–1^) increased survival to 26 days (*P* < 0.0001), and the association of the drugs (3H2 30 mg kg^–1^ + AMB 0.5 mg kg^–1^) prolonged survival for more than 100 days (*P* < 0.0001). After the treatment was ceased, the animals were monitored for an additional 30 days and then euthanized to analyze their fungal burden.

**Figure 7:**
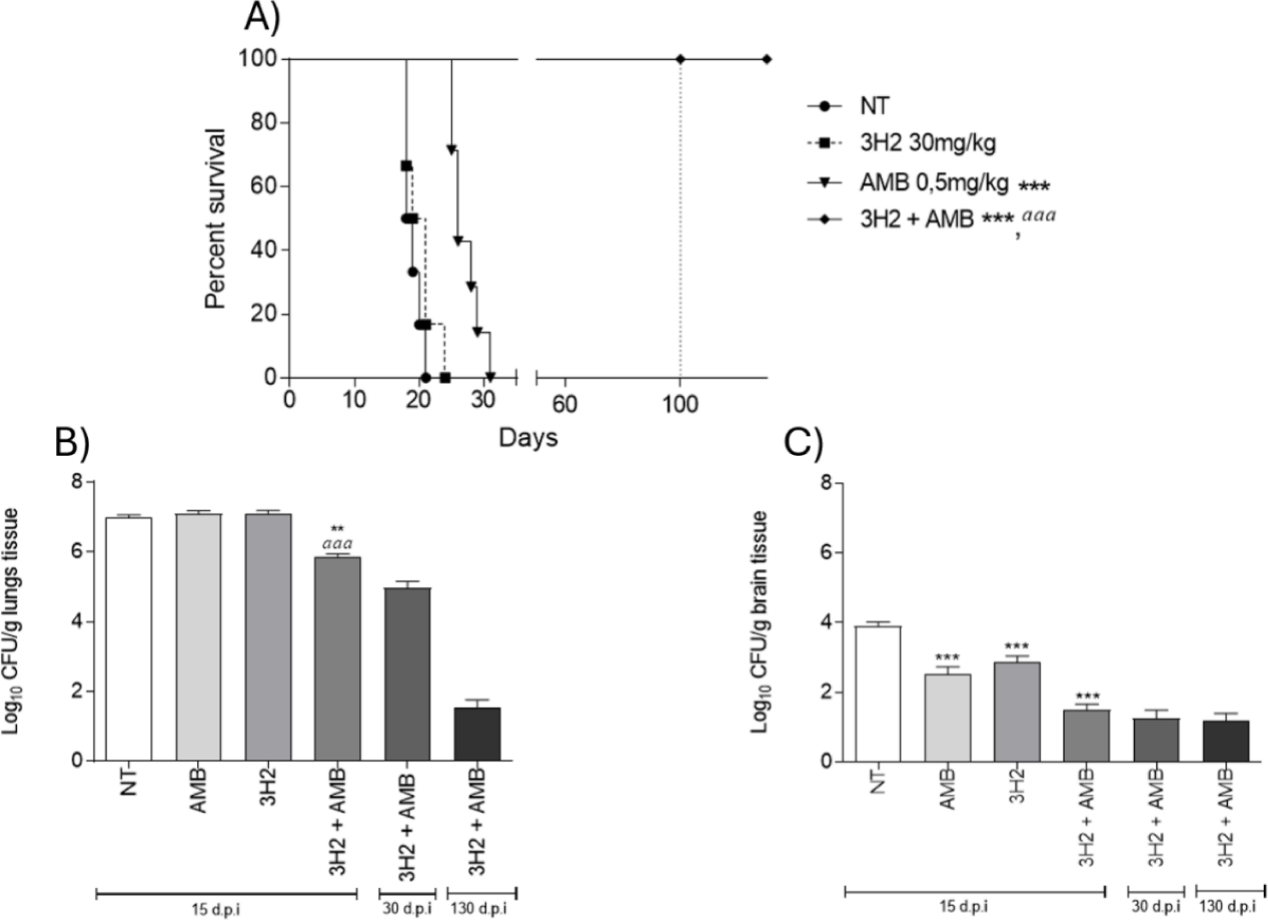
Compound 3H2, when associated with amphotericin B (AMB), increases survival and reduces fungal burden in mice inoculated with *C. gattii* L27/01. survival curve of mice infected with *C. gattii* without treatment (NT) and treated with 3H2 and AMB alone and in combination is shown over 100 days of treatment, with the animals monitored for more than 30 days (A). Fungal burden in the brains (B) and lungs (C) of the same groups was assessed after 15, 30, and 130 days post-infection (d.p.i). *P < 0.05; **P < 0.001;***P < 0.0001 when compared with the NT group; *^aaa^P* < 0.0001 when compared with AMB in monotherapy. *n* = 6 mice per group.

All treatments reduced the fungal burden in the brains of mice in comparison with the NT group (*P* < 0.0001). It is noteworthy that even after 100 days of treatment and 30 days of monitoring (130 days post-infection), there was still detection of the yeast in this organ. This was also observed in the lungs of mice treated with 3H2 + AMB (Figure 7B). Besides, after 15 days of infection, only the association of the drugs reduced the fungal burden in the lungs (*P* < 0.001), being even more effective than AMB (*P* < 0.001) (Figure 7C).

The SHIRPA protocol was performed to evaluate behavioral aspects that might change due to cryptococcosis or the toxic effects of amphotericin B (Figure 8). There were no differences between groups in the analysis of sensorial reflex and autonomous function. The neuropsychiatric state was affected in the group treated with AMB, and the combination treatment showed a better score (*P* < 0.0001). There was an improvement in the motor behavior of animals treated with 3H2 + AMB compared to those treated with AMB only (*P* < 0.0001). The muscle tone of animals treated with the association of AMB and 3H2 showed better scores when compared to those not treated or treated only with AMB (*P* < 0.001). In maintaining the body weight of mice, it was also observed that the combination was more effective than the monotherapies used (*P* < 0.05).

**Figure 8:**
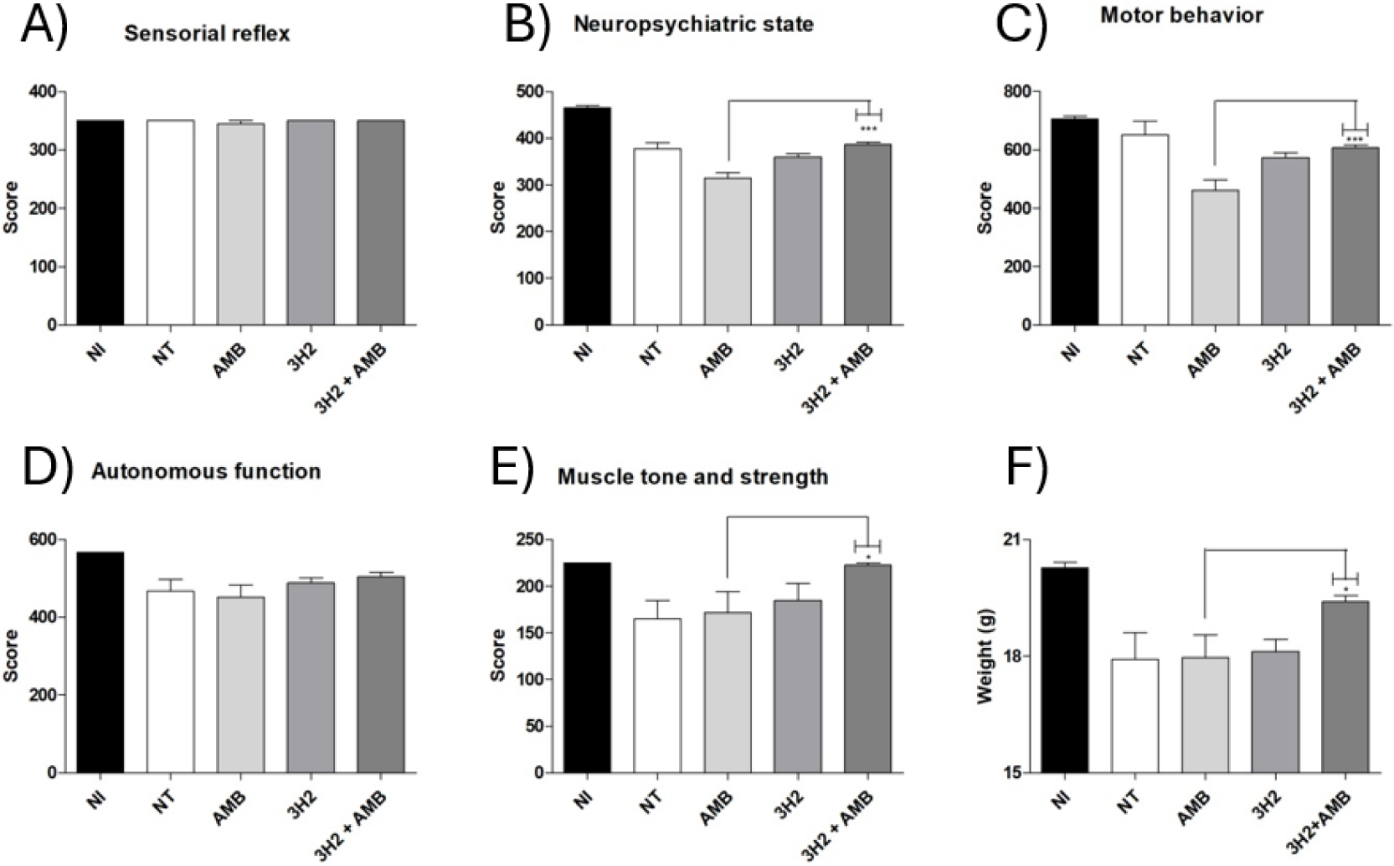
Behavioral profile evaluation (SHIRPA protocol) of animals infected with *C. gattii* and treated with 3H2, AMB alone, or the combination. Parameters assessed included sensorial reflex (A), neuropsychiatric state (B), motor behavior (C), autonomous function (D), muscle tone and strength (E) and body weight (F). *P < 0.05; **P < 0.001; ***P < 0.0001. The connected lines indicate differences between AMB alone and AMB + 3H2.

### 3.5 Docking Study

Molecular docking simulations were performed using the crystallographic structure of a fungal laccase from *Trametes versicolor* (PDB: 1KYA), due to the absence of an experimentally resolved structure for *Cryptococcus gattii* laccase. To evaluate the structural relevance of this template, a pairwise BLASTp alignment was performed between *C. gattii* multicopper oxidase (UniProt Q49TP1) and the 1KYA sequence. The alignment revealed 28.9% sequence identity with 84% query coverage and a highly significant E-value (5 × 10^–57^), indicating statistically meaningful homology within the multicopper oxidase family. Conserved residues associated with copper coordination were located within aligned regions, supporting the preservation of the catalytic architecture. Site 1 was defined based on the coordinates of the cocrystallized ligand, whereas Site 2 was defined by spatial constraints encompassing the solvent-accessible region adjacent to the T2/T3 cluster.

Docking analysis showed that compound 3H2 can establish relevant interactions in two structural regions of laccase. In site 1, corresponding to the type 1 copper center (T1),^38^ 3H2 adopts a well-defined and strongly supported binding mode. The phenolic hydroxyl group forms hydrogen bonds with Asn-264 (2.5 Å) and Asp-206 (2.3 Å), contributing to consistent polar anchoring near the electron-transfer site. In addition, a structured set of hydrophobic and aromatic interactions is observed, including π–π stacking with Phe-239 (4.2 Å) and Phe-162 (4.0 Å), and further hydrophobic contacts with Phe-265 (4.1 Å), Ile-455 (4.6 Å), and Leu-164 (4.0 Å). Together, these interactions support favorable and complementary accommodation at the T1-associated pocket, identifying this region as the primary binding site for the ligand (Goldscore: 17.5703) (Figure 9).

**Figure 9:**
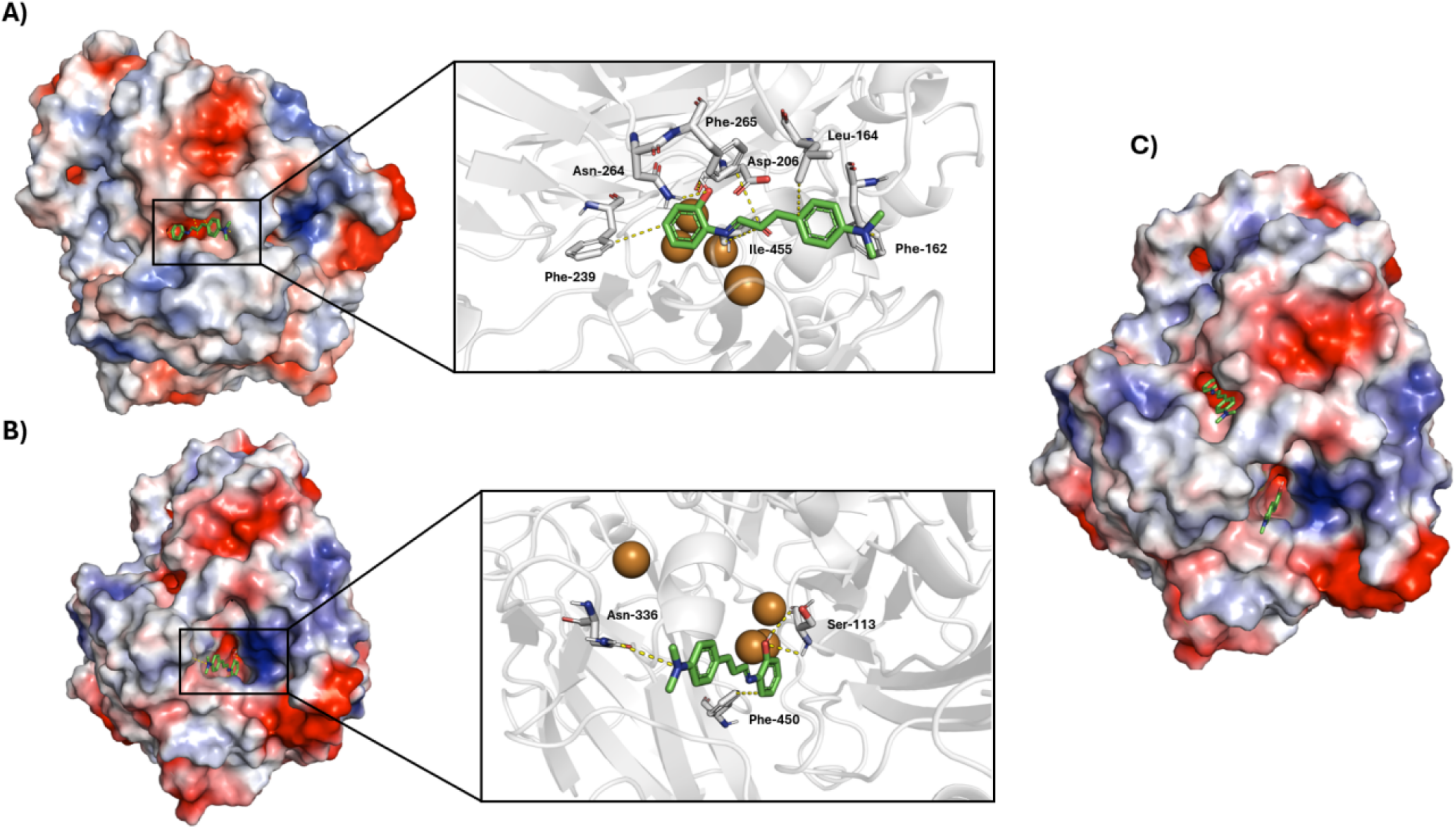
Molecular docking of 3H2 is illustrated. (A) Docking pose of 3H2 at laccase binding site 1. (B) Docking pose of 3H2 at laccase binding site 2. (C) Surface representation of laccase showing both binding sites interacting with 3H2 (PDB ID: 1KYA).

In site 2, corresponding to the accessible region adjacent to the trinuclear T2/T3 copper cluster,^38^ an alternative binding mode is observed. Because the T2/T3 cluster is structurally buried, molecules of 3H2 size tend to interact only with peripheral residues in this region. The phenolic hydroxyl group forms two hydrogen bonds with Ser-113 (2.3 and 2.8 Å), and the dimethylamino group establishes contact with Asn-336 (4.1 Å). An additional aromatic interaction with Phe-450 (3.7 Å) contributes modestly to ligand stabilization. However, the reduced number of interactions and the weaker spatial complementarity indicate that this pocket acts as a secondary binding site, structurally less favored compared with T1, as evidenced by an unfavorable fitness score (−44.8811).

Thus, although docking suggests the possibility of two binding modes, the interaction profiles clearly indicate that T1 is the primary binding site for 3H2, while the region associated with the T2/T3 cluster functions as a secondary site of lower functional relevance. These findings reinforce the predominance of T1 as the main recognition region for aromatic compounds and suggest that 3H2 may explore secondary microenvironments near the trinuclear center, albeit with reduced stability.

Docking results indicate that 3H2 preferentially binds to the T1 site, supported by a coherent network of polar and aromatic interactions. In contrast, the peripheral region near the T2/T3 cluster acts as only a secondary site, offering fewer and weaker contacts. These findings reinforce T1 as the primary recognition site for 3H2, while the T2/T3 region contributes only marginally to ligand stabilization.

## 4 Conclusions

This study demonstrates that allylimine derivative 3H2 displays relevant anticryptococcal activity against *Cryptococcus gattii* through multiple complementary mechanisms. In addition to inhibiting fungal growth *in vitro*, the compound significantly modulated key virulence determinants, including capsule size, melanization, and cell surface charge, suggesting interference with biological processes associated with pathogenicity.

Although 3H2 alone did not substantially extend survival in the murine model, it produced a consistent reduction in fungal burden in the brain and exhibited a marked, synergistic interaction with amphotericin B. This combination resulted in prolonged survival, lower fungal loads in the lungs and brain, and improved clinical parameters in infected animals, highlighting the therapeutic potential of this association.

Molecular docking analysis provided structural support for these observations by suggesting favorable interactions between 3H2 and the type-1 copper region of laccase, a key enzyme involved in melanin biosynthesis and fungal virulence. Taken together, these findings indicate that 3H2 may act not only as a direct antifungal agent but also as a modulator of virulence-associated pathways.

Overall, the present work provides the first *in vivo* evidence supporting the anticryptococcal potential of this allylimine scaffold. Considering the limited therapeutic options currently available for cryptococcosis and the benefits observed in combination therapy, 3H2 represents a promising starting point for further pharmacological optimization and mechanistic investigation as an adjunctive antifungal strategy.

## Supplementary Material



## Notes

Experimental procedure for the synthesis of 3H2; ^1^H and ^13^C NMR spectroscopic data and spectra (Figures S1–S2); computational methods and geometry optimization; molecular docking protocol; redocking validation of laccase (PDB ID: 1KYA); scoring function comparison and RMSD analysis (Table S1); time–kill kinetics assay against *Cryptococcus gattii* (Figure S3) (PDF)
