# Supplementary material for: Anticryptococcal Evaluation of the Allylimine 3H2: Modulation of Virulence Traits and Synergistic Action with Amphotericin B
Source: ACS Omega. 2026 Apr 27;11(18):27183–92. doi: 10.1021/acsomega.6c01027 (PMC13177235; doi:10.1021/acsomega.6c01027)
Supplement: Supplementary file 1 [file ao6c01027_si_001.pdf]

Supplementary Material

Experimental procedure for synthesis of **3H2**; ^{1}H and ^{13}C NMR spectroscopic data and spectra (Figures S1–S2); computational methods and geometry optimization; molecular docking protocol; redocking validation of laccase (PDB ID: 1KYA); scoring function comparison and RMSD analysis (Table S1); time–kill kinetics assay against *Cryptococcus gattii* (Figure S3).

Chemistry

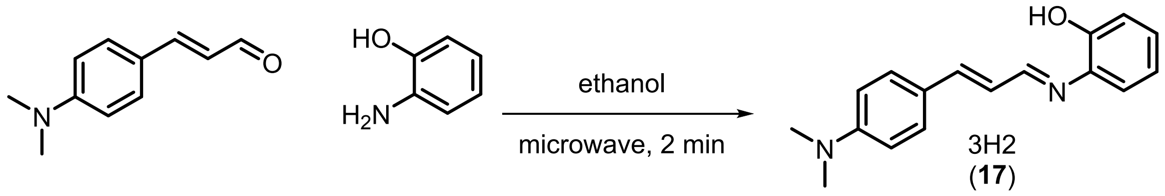

Compound 3H2 was obtained through the condensation of 4-dimethylaminocinnamaldehyde with *O*-hydroxyaniline under microwave irradiation, leading to the formation of a Schiff base. Equimolar amounts of both reagents were dissolved in absolute ethanol and irradiated for 2 min in a CEM Discover microwave reactor. The reaction progress was followed by thin-layer chromatography. After completion, the reaction mixture was cooled to room temperature, and the crude material was purified by crystallization from ethanol to afford the allylimine product in 80% yield.

^{1}H NMR (400 MHz, DMSO-d_6) δ 8.77 (s, 1H), 8.37 (d, J = 9.0 Hz, 1H), 7.48 (d, J = 8.8 Hz, 2H), 7.21 (d, J = 15.8 Hz, 1H), 7.06 (dd, J = 7.8, 1.5 Hz, 1H), 7.01 (td, J = 8.0, 1.6 Hz, 1H), 6.90 (dd, J = 15.8, 9.0 Hz, 1H), 6.86 – 6.76 (m, 2H), 6.74 (d, J = 8.9 Hz, 2H), 2.98 (s, 6H).

^{13}C NMR (100 MHz, DMSO) δ 161.29, 151.15, 150.89, 144.42, 138.71, 128.96, 126.53, 123.72, 123.12, 119.44, 118.76, 115.64, 112.01.

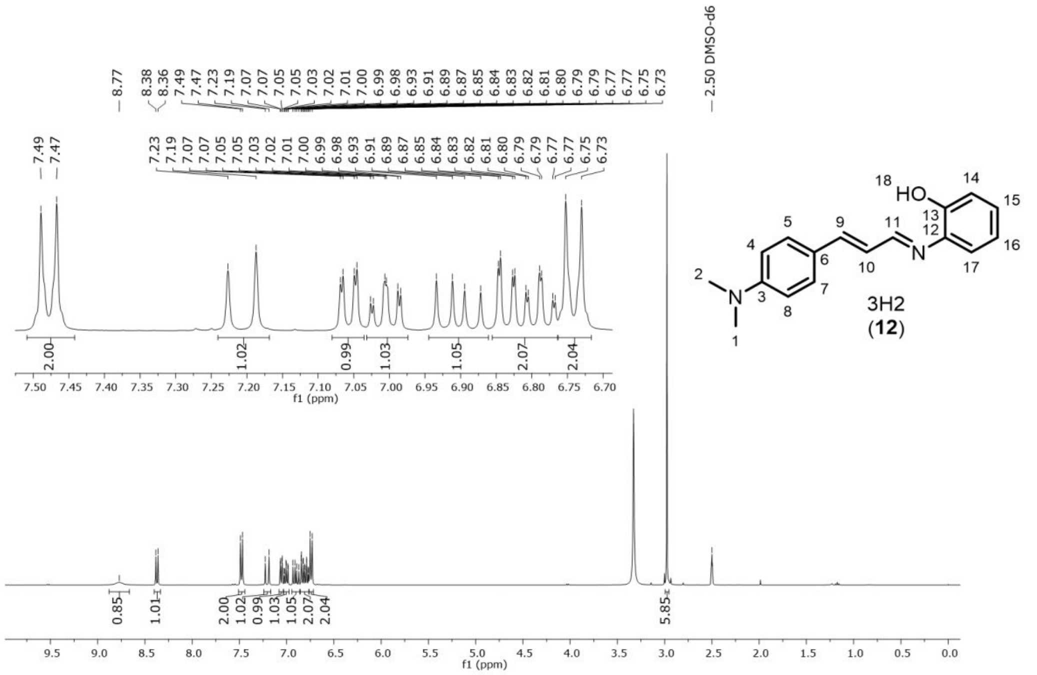

Figure S1 – ^{1}H NMR spectrum (400 MHz, DMSO-d_6) of compound 3H2.

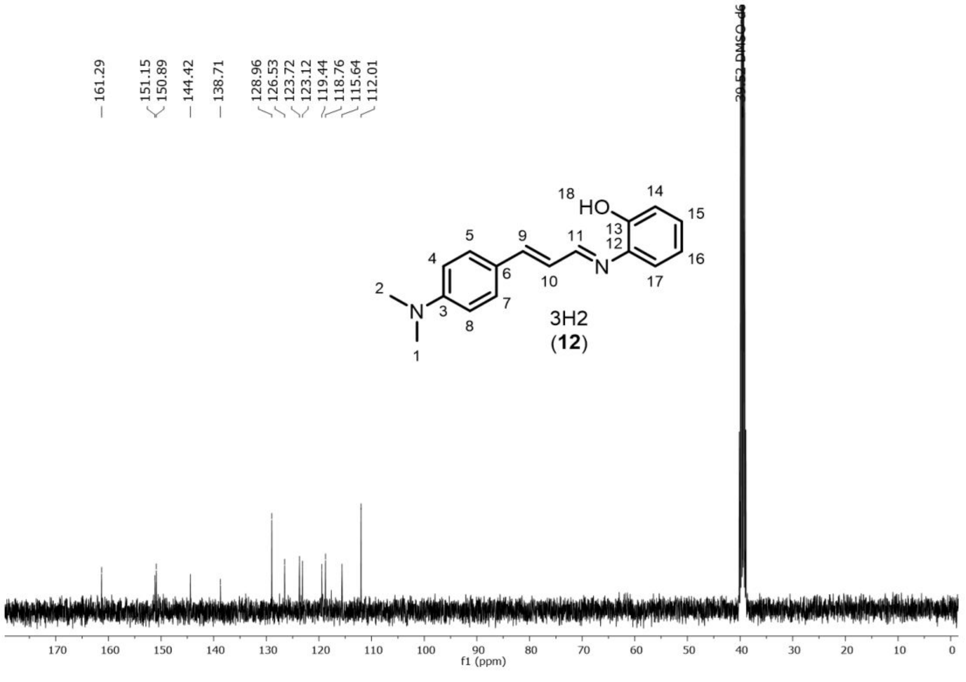

Figure S2 – ^{13}C NMR spectrum (100 MHz, DMSO-d_6) of compound 3H2.

Validation – Redocking

The initial three-dimensional structures of the molecules were constructed using Avogadro software (version 1.2.0) and subsequently subjected to geometry optimization by density functional theory (DFT) calculations employing the ORCA package (version 6.0.1). Molecular docking studies were conducted using the crystallographic structure of active laccase from trametes versicolor retrieved from the Protein Data Bank (PDB ID: 1KYA; resolution: 2.40 Å). This structure contains a co-crystallized ligand, 2,5-dimethylaniline,

which was used as a reference to delimit the binding region. Docking simulations were performed with the GOLD software (version 2022.3.0, CCDC; License key: G/4142006). Hydrogen atoms were added to the protein using GOLD's built-in preparation tools. The binding pocket was defined by selecting all amino acid residues with atoms located within a 10 Å radius of the reference ligand.To determine the most appropriate scoring function for the docking protocol, a validation step was carried out by re-docking the native ligand after removal of crystallographic water molecules. Four scoring functions implemented in GOLD (GoldScore, ChemScore, ChemPLP, and ASP) were assessed by calculating the root-mean-square deviation (RMSD) between the experimentally observed ligand pose and the top-ranked docked conformation. GoldScore demonstrated superior performance, based on both RMSD values and the average scores of the ten highest-ranked poses (**Table S1**), and was therefore selected for subsequent semi-flexible docking studies of the investigated compounds. For the ligand, three independent docking calculations were conducted, each producing ten distinct poses. From each run, the highest-scoring pose was retained for further evaluation. Final pose selection considered not only the numerical docking scores but also the structural consistency and chemical plausibility of the predicted protein–ligand interactions.

Table S1: Superposition of co-crystallized and re-docked ligand with GOLD 2022.3.0 program. RMSD = 1.30313 Å.

**Redocking - LACCASE (PDB ID: 1KYA) Resolution: 2.40 Å**			
**GoldScore**	ChemScore	ChemPLP	ASP
**1.30313** 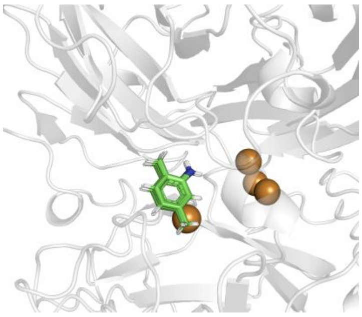	1.47146	2.04812	3.99761

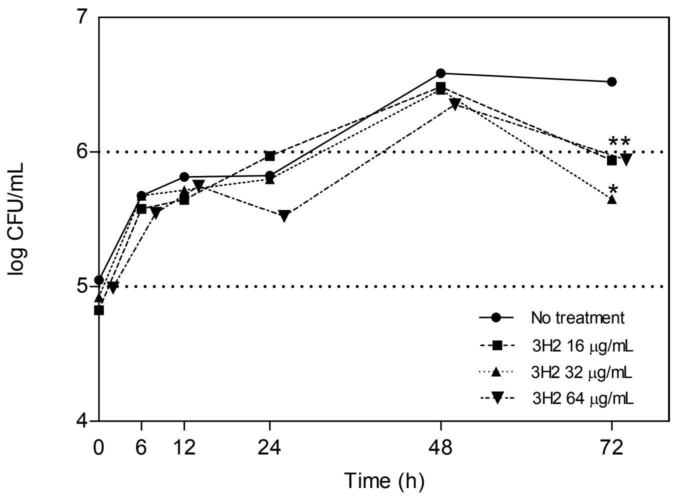

Figure S3: Time-kill kinetics of *Cryptococcus gattii* (strain L27/01) treated with 3H2. Growth curve illustrating the viable cell counts (Log10 CFU/mL) over a 72-hour period (0, 6, 12, 24, 48, and 72 h) in the absence of treatment (Control) and in the presence of 3H2 at 16, 32, and 64 μgmL^{-1}. Asterisks (*) indicate a statistically significant reduction in cell counts at 72 h compared to the control ($P < 0.05$).
